# Intracellular role of IL-6 in mesenchymal stromal cell immunosuppression and proliferation

**DOI:** 10.1038/s41598-020-78864-4

**Published:** 2020-12-14

**Authors:** Akaitz Dorronsoro, Valérie Lang, Izaskun Ferrin, Jon Fernández-Rueda, Lorea Zabaleta, Estibaliz Pérez-Ruiz, Pilar Sepúlveda, César Trigueros

**Affiliations:** 1 Mesenchymal Stem Cell Laboratory, Fundación Inbiomed, Foundation for Stem Cell Research, Paseo Mikeletegi, 81, 20009 San Sebastián, Spain; 2 Regenerative Medicine and Heart Transplantation Unit, Instituto de Investigación Sanitaria La Fe, Avenida Fernando Abril Martorell, 106, Torre A, Laboratorio 5.04, 46026 València, Spain

**Keywords:** Cytokines, Inflammation, Cell division, Cell signalling

## Abstract

Interleukin (IL)-6 is a pleiotropic cytokine involved in the regulation of hematological and immune responses. IL-6 is secreted chiefly by stromal cells, but little is known about its precise role in the homeostasis of human mesenchymal stromal cells (hMSCs) and the role it may play in hMSC-mediated immunoregulation. We studied the role of IL-6 in the biology of bone marrow derived hMSC in vitro by silencing its expression using short hairpin RNA targeting. Our results show that IL-6 is involved in immunosuppression triggered by hMSCs. Cells silenced for IL-6 showed a reduced capacity to suppress activated T-cell proliferation. Moreover, silencing of IL-6 significantly blocked the capacity of hMSCs to proliferate. Notably, increasing the intracellular level of IL-6 but not recovering the extracellular level could restore the proliferative impairment observed in IL-6-silenced hMSC. Our data indicate that IL-6 signals in hMSCs by a previously undescribed intracellular mechanism.

## Introduction

Mesenchymal stromal cells (MSCs) are multipotent progenitor cells with the capacity to self-renew and to differentiate into multiple lineages. MSCs can be isolated from a variety of tissues, although bone marrow is the most common source for research and clinical purposes. MSCs are attractive cell candidates for tissue engineering applications, in particular for bone and cartilage repair due to their ability to differentiate into the chondrocyte, osteoblast or adipocyte lineages^1,2^. Many studies have described that MSCs exert beneficial effects when administered during a narrow therapeutic window, and their immunomodulatory features and capacity to home to damaged tissues have placed MSCs in the spotlight as advanced therapies for a broad range of autoimmune disorders^3–5^. The precise mechanisms by which MSCs regulate immune functions are still not fully understood, although many studies have described different soluble factors to be involved in these processes, including indoleamine 2,3-dioxygenase (IDO), prostaglandin E2 (PGE2), transforming growth factor-β, hepatocyte growth factor, interleukin (IL)-10, and the human leukocyte antigen-G^6^. It is known that the inflammatory environment in which MSCs are exposed after infusion is a critical determinant of their regulatory process, as immunosuppression by MSCs is not constitutive but rather triggered by crosstalk with cells of the immune system^7–10^. Accordingly, different pathological scenarios will lead to distinct responses from MSCs, which should be considered when designing clinical interventions, together with treatment dose, timing and frequency of administration, as well as the source of MSCs.

We previously demonstrated that human MSCs (hMSCs) modulate T-cell responses through TNF-α-mediated activation of NF-κB, underlining the importance of this transcription factor in the immunomodulatory capacity of hMSCs^11^. To better understand the involvement of NF-κB in this process, here we studied the role of one of the most important NF-κB targets, IL-6.

IL-6 is often referred to as a pleiotropic cytokine that acts on numerous cell types and influences multiple biological activities. IL-6 can be synthesized and secreted by many cell types including monocytes, T-cells, fibroblasts and endothelial cells^12^. Binding of IL-6 to its cognate membrane-bound receptor IL-6R triggers the binding of a second trans-membrane protein, gp130, which serves as a signal transducer of IL-6. While gp130 is expressed on all cells of the body^13^, the expression of IL-6R is restricted to hepatocytes, neutrophils, monocytes and CD4+ T-cells^14,15^. In the past few years, a critical role for IL-6 has been demostrated in numerous inflammatory diseases (reviewed in^12^). IL-6 can signal through two distict pathways, known as classic- and trans-signaling. Whereas classic signaling via membrane-bound IL-6R is considered mostly protective and regenerative, the alternative IL-6 “trans”-signaling pathway through a soluble form of the IL-6 receptor (abundant in extracellular fluids) is believed to represent a stress response of the body to maintain homeostasis^15^. The role of IL-6 in the regulation of the immune system has been described both as pro-inflammatory and anti-inflammatory. For instance, IL-6 induces endothelial permeability and cell recruitment, as well as B-cell maturation and T-cell survival^16–18^. However, it is also involved in the secretion of well-known anti-inflammatory molecules, such as the IL-1 receptor antagonist or IL-10, and reduces the abundance of TNF-α. A better understanding of the regulation of IL-6 signaling is fundamental to comprehend both the physiological and the pathophysiological functions of this pleiotropic cytokine, and to develop therapeutic strategies exploiting its properties^12,19^.

In the present work, we explored the involvement of IL-6 in the immunomodulatory capacity of hMSCs using lentiviral-mediated short hairpin RNAs (shRNAs) to silence its expression. We found that silencing of IL-6 diminished the ability of hMSCs to suppress T-cell proliferation. At the same time, we observed that IL-6 silencing has no impact on hMSC survival but reduces cell proliferation by blocking the progression of the cell cycle. Finally, our data strongly suggest that IL-6 regulates these processes through an intracellular signaling pathway, highlighting the importance of this intracellular mechanism over the extracellular effects of IL-6 on hMSC physiology.

## Materials and methods

### Human samples

Human bone marrow-derived MSCs (hMSCs) were obtained from the Inbiobank Stem Cell Bank (www.inbiobank.org) as described^20^. Briefly, cadaveric bone marrow was harvested from brain-dead donors after informed consent and under the supervision of the Spanish National Organization of Transplants (in Spanish, *Organizacion Nacional de Transplantes*). The study was approved by the Clinical Research Ethical Committee of the *Hospital Donostia* (Donostia, Spain), and all procedures were carried out in accordance with Spanish law (14/2007) on biomedical research and the Royal Decree 1716/2011 regulating activities related to the use of human tissues in Spain. Generated hMSCs display a typical CD29+ , CD73+ , CD90+ , CD105+ , CD166+ , CD146+ , CD34− , CD45− , CD14− , CD19− and CD31− phenotype; a fibroblast-like morphology; and at least tri-lineage potential, including osteocyte, chondrocyte and adipocyte generation^21^. hMSCs were cultured in low-glucose DMEM (Sigma-Aldrich, Madrid, Spain) supplemented with 10% FBS (Fisher Scientific, Madrid, Spain). On reaching confluence, hMSCs were collected with trypsin and seeded at 1 × 10^3^ cells/cm^2^. Cells were obtained at passage three from the Stem Cell Bank and all experiments were performed with cultures at passage 4 to 8. Cells were passaged when they reached 75% confluency to avoid excessive cell density. When indicated MSC were treated with TNF-α (R&D Systems, Minneapolis, MN, 210-TA).

Blood samples and data from patients included in this study were provided by the Basque Biobank for Research-OEHUN (www.biobancovasco.org) and were processed following standard operating procedures with appropriate approval of the local Ethical and Scientific Committees. Peripheral blood mononuclear cells (PBMCs) were purified from buffy coats by density gradient using Lymphoprep (ATOM, Barcelona, Spain). PBMCs were frozen for preservation until use.

### Cell culture

PBMCs were stimulated with Dynabeads Human T-Activator CD3/CD28 (Life Technologies, Foster City, CA) plus IL-2 (10 ng/ml, R&D Systems), as described^11^. A ratio of 1:1 of CD3/CD28 beads to PBMCs was used, as recommended by the manufacturer. PBMCs (250,000 cells) were cultured in RPMI medium supplemented with 10% FBS in the presence or absence of hMSCs (10,000 cells) during 6 days. Expansion indices were calculated with FlowJo analysis software (Treestar Inc., Ashland, OR). When indicated, cells were treated with dexamethasone (Sigma-Aldrich, 1 nM), indomethacin (Sigma-Aldrich, 5 μM), etoricoxib (Sigma-Aldrich, 5 μM), recombinant human IL-6 (rhIL-6; R&D Systems, 206-IL) or an anti-IL-6 neutralizing antibody (eBioscience, San Diego, CA7069-85).

### Transduction of shRNAs

shRNA expression vectors were constructed using standard cloning procedures. The following shRNA sequences have been published previously^22^ and were purchased from Sigma-Genosys (Oakville, ON, Canada): IL-6ia: AGATGGATGCTTCCAATCTGG and IL-6ib: AAGGCAAAGAATCTAGATGCA. Both targeting sequences were purchased from the RNAi Consortium (www.broadinstitute.org/rnai). We used two different target sequences to avoid off-target effects. Oligonucleotides were annealed and cloned into the pSUPER plasmid carrying an H1 promoter using BglII–HindIII sites. The H1-shRNA expression cassette was then excised and cloned into pLVTHM (Addgene plasmid 12,247, www.addgene.org) using EcoRI–ClaI sites^21^. Viral particles were produced as described by the Viral Vector Platform at Inbiomed Foundation^21^. hMSC transduction was carried out at a multiplicity of infection of ten in order to achieve 100% infection. When indicated, transduction was performed to obtain 50% infection to compare from the same population the effect of infection on GFP+ and GFP- cells.

### Flow cytometry

Cell cycle analysis was performed as described Briefly, hMSCs were fixed and washed twice with PBS and resuspended in PBS containing 5 mg/ml propidium iodide (PI) and 10 μg/ml RNase A (Sigma-Aldrich). Cell cycle analysis was performed on GFP (530/30BP emission filter)-positive and living cells, excluding doublets^23^.

IL-6 levels were measured in samples with a custom cytometric bead array kit (CBA; BD Biosciences, San Jose, CA) for IL-6 following the manufacturer’s instructions^11^. Samples were incubated with the CBA during 30 min and were mixed with the combined cocktail of phycoerythrin (PE)-conjugated antibodies. IL-6 concentration was measured via quantification of PE fluorescence in reference to a standard curve.

Apoptosis was evaluated by flow cytometric determination of Annexin-V DY634 (Immunostep, Salamaca, Spain) staining on GFP (530/30BP)-positive cells, excluding doublets^24^. Briefly, hMSCs were treated overnight with IL-6 (10 ng/ml, R&D Systems, 206-IL), stained with Annexin-V DY634 in 1 × binding buffer (10 mM HEPES [pH 7.4], 140 mM NaOH, 2.5 mM CaCl_2_) for 15 min at room temperature in the dark and analyzed on a FACSCanto flow cytometer (BD Biosciences) using FlowJo software (www.flowjo.com). Data represent the mean of three independent experiments performed in triplicate.

### Expression analysis

Total RNA was extracted using the RNAeasy Extraction Kit (Qiagen, Hilden, Germany). cDNA was obtained using the GeneAmp Reverse Transcriptase Kit (Applied Biosystems, Foster City, CA). Quantitative PCR was performed using the Power SYBRR Green PCR Master Mix (Applied Biosystems). *IL-6*, *IDO*, *COX2* and *GAPDH* were amplified using the following oligonucleotide pairs: *IL-6*–AACGCTCCTCTGCATTGCCATT and GAGCAGCCCCAGGGAGAA; *IDO*–CTACCATCTGCAAATCGTGACTAAG and GAAGGGTCTTCAGAGGTCTTATTCT; *COX2*–GAATCATTCACCAGGCAAATTG and TCTGTACTGCGGGTGGAACA; *GAPDH*–TGCACCACCAACTGCTTAGC and GGCATGGACTGTGGTCATGAG. Reactions were carried out in a Step One Plus Thermocycler (Applied Biosystems). Data were compared using the comparative CT method, normalizing all samples against hMSCs infected with the empty vector control (pLVTHM emp). *GAPDH* was used as a housekeeping gene control.

For western blot analysis, 1 × 10^5^ infected cells were stimulated or not with TNF-α (15 ng/ml) or IL-6 (20 ng/ml) and lysed, as described^25^. Proteins were then separated in 10% w/v SDS–polyacrylamide gel (SDS-PAGE) and subsequently transferred onto polyvinylidene difluoride membrane. Immunodetection was performed with the following primary antibodies: IL-6, (rabbit), cyclin D1 (mouse) (both from Santa Cruz Biotechnology, Sant Cruz, CA), ERK1/2, phospho-ERK1/2 (BD biosciences, Mouse) and GAPDH (Sigma-Aldrich, Mouse). Secondary antibodies were HRP-conjugated sheep anti-rabbit IgG or HRP-conjugated sheep anti-mouse IgG (GE Healthcare Amersham, Little Chalfont, UK).

### Detection of prostaglandin E2

PGE2 levels were determined using a commercial ELISA kit (R&D Systems) on supernatants of hMSC cells transduced with pLVTHM emp or pLVTHM IL-6i, treated or not, as indicated, with indomethacin or etoricoxib for 48 h.

### Immunofluorescence

To determine the intracellular localization of p65 or KI-67, cells were fixed with 4% paraformaldehyde solution (Pancreac, Barcelona, Spain) for 10 min and permeabilized with PBS-Triton X-100 (0.1%) for 10 min. Staining was performed using a rabbit anti-human p65 (Santa Cruz Biotechnology) or KI-67 (BD Biosciences) antibody, and revealed with a donkey anti-rabbit IgG secondary antibody conjugated with Cy-3 (Jackson Immunoresearch, West Grove, PA). Analyses were performed using an LSM 510 Meta Laser Scanning Microscope (Zeiss, Jena, Germany) at the Cytometry and Advanced Microscopy Platform at the Inbiomed Foundation.

### Statistical analysis

Data are expressed as mean ± standard error of the mean. Student’s t test was used for comparison between groups. When the distribution was not normal, the Mann–Whitney U test was used. Analysis of variance and the Kruskal–Wallis test were used to compare the means of more than 3 groups. Analyses were conducted with GraphPad Prism 8 software (GraphPad Software Inc., La Jolla, CA). Differences were considered statistically significant at *p* < 0.05 with a 95% confidence interval.

### Ethics approval and consent to participate

The use of human cells and the project were approved by the Ethical committee of Inbiobank.

## Results

### IL-6 is involved in the immuregulatory function of hMSCs

**Figure 1:**
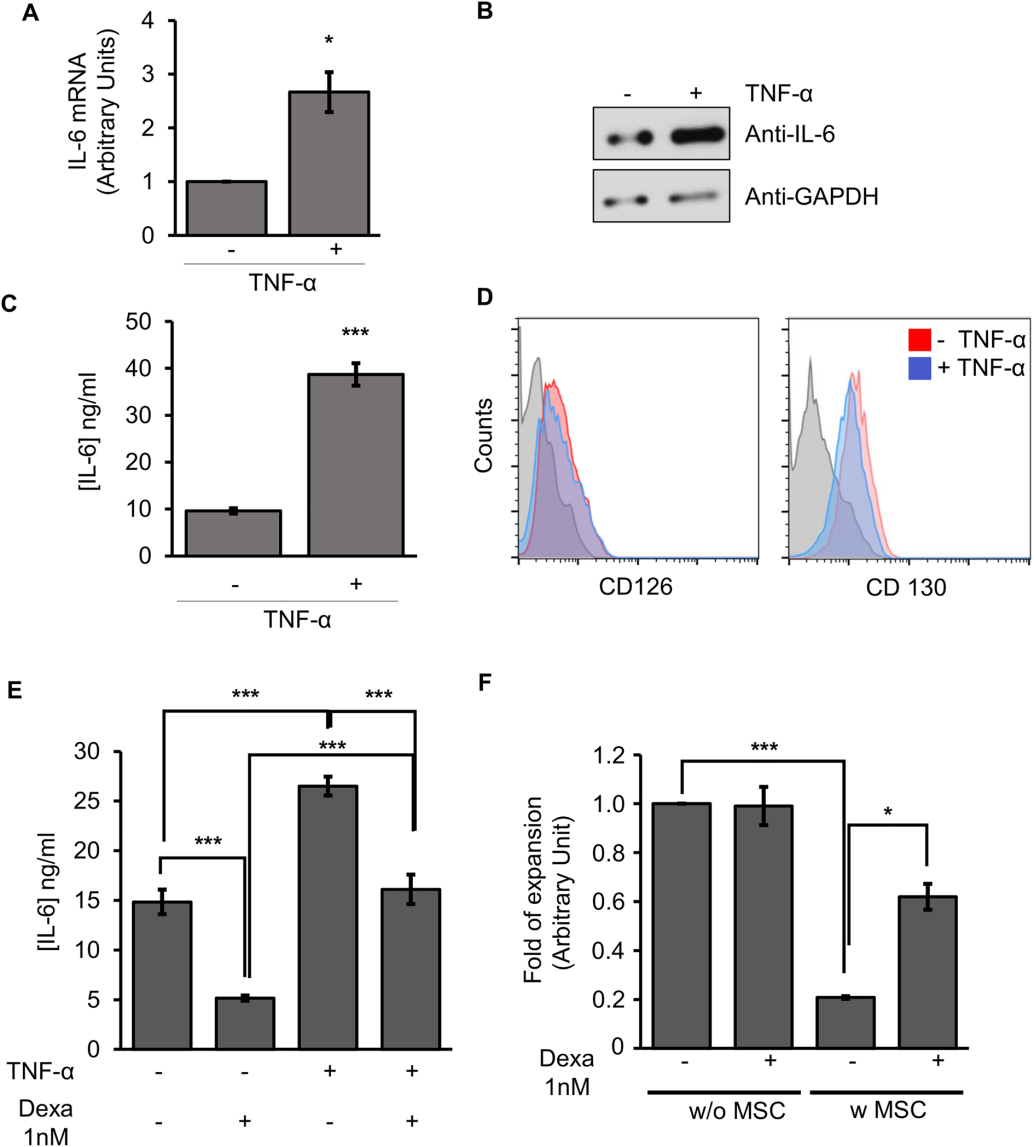
IL-6 secretion by hMSCs can be modulated by TNF-α and dexamethasone. (**A**) Quantification of *IL-6* mRNA by real time qPCR. The expression levels of the target gene were normalized against *GAPDH* expression. (**B**) Western blot using an anti-IL-6 antibody. (**C**) IL-6 secretion determined using a CBA kit in hMSCs treated or not with TNF-α (15 ng/ml) for 48 h. Columns represent mean values of three independent experiments, and error bars represent the mean ± SD of these experiments. *p < 0.05, *** *p* < 0.001. (**D**) hMSCs expressed the IL-6 receptors CD126 and CD130 in absence or presence of TNF-α during 48 h. Quantification of IL-6R (CD126), CD130 using flow cytometry. Filled histograms represents isotype antibody, red histogram represents MSC not treated with TNF-α and blue histogram represents MSC treated with TNF-α. (**E**) Inhibition of IL-6 secretion in cell culture supernatant of hMSCs treated with or without dexamethasone (1 nM) and TNF-α (15 ng/ml). ***p < 0.001 (**F**) Proliferation of T-cells cultured with (w) or without (w/o) hMSC in the presence or absence of dexamethasone (1 nM).

We previously demonstrated that TNF-α/NF-κB priming/signaling regulates immunoregulatory profile of hMSCs, which could be inhibited by the presence of glucocorticoids such as dexamethasone^11^. Intrigued by the mechanisms involved in the modulation of hMSC properties, we focused our attention on IL-6, whose expression is under the control of NF-κB. Although hMSC constitutively express IL-6, TNF-αinduced a marked and statistically significant increase in IL-6 expression at the level of mRNA (Fig. 1A), protein (Fig. 1B) and secretion (Fig. 1C). Additionally, both alpha and beta subunits of IL-6R (CD126 and CD130, respectively) were also expressed in hMSC (Fig. 1D), suggesting that the IL-6 could have an autocrine role in the biology of these cells. Signalling with TNF-α priming did not significantly alter the abundance of the receptors in the membrane of hMSCs (Fig. 1D). Dexamethasone treatment of hMSCs inhibited both basal and TNF-α-induced IL-6 secretion (Fig. 1E), which correlated with a reduced capacity of hMSCs to impact T-cell proliferation (Fig. 1F).

**Figure 2:**
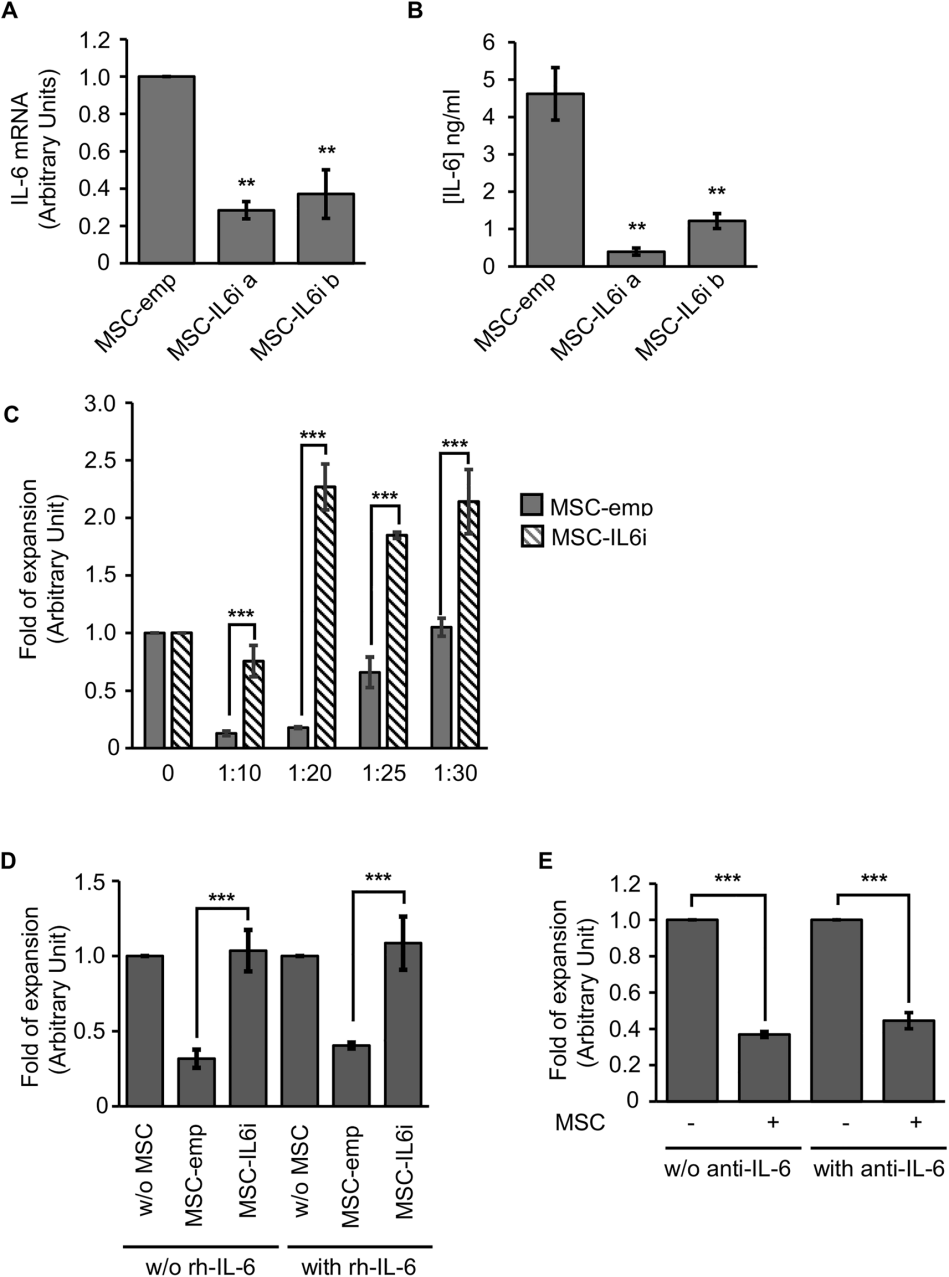
IL-6 is involved in hMSC-mediated inhibition of activated T-cell proliferation. (**A**) Quantification of IL-6 expression by real time qPCR. Graphs represent mean values of three independent experiments, and error bars represent the mean ± SD of the experiments. The expression levels of the target gene were normalized against GAPDH expression. **p < 0.01. (**B**) Quantification of IL-6 secretion using a CBA kit. Graphs represent mean values of three independent experiments, and error bars represent the mean ± SD of these experiments. **p < 0.01. (**C**) Proliferation of T-cells cultured in the absence of hMSCs (0), co-cultured with hMSC-emp or hMSC-IL6i using different ratios of hMSCs/T-cells. Data are shown as mean ± SD from three independent experiments. ***p < 0.001. (**D**) Proliferation of T-cells cultured in the absence of hMSCs, (w/o), co-cultured with hMSC-emp or hMSC-IL6i in the absence (w/o) or presence of recombinant IL-6 (20 ng/ml). Data are shown as mean ± SD from three independent experiments. (**E**) Proliferation of T-cells cultured in the absence of hMSCs or co-cultured with hMSC, in presence or absence (w/o) of a blocking anti-IL6 antibody (5 μg/ml). Data are shown as mean ± SD from three independent experiments. **p < 0.01.

To test whether the effect of dexamethasone on the hMSC immunoregulatory function was mediated by the decrease in IL-6 expression, we used a GFP-expressing lentiviral vector to transduce more than 95% of hMSCs with an empty vector or a shRNA targeting IL-6 (hereafter referred to as hMSC-emp and hMSC-IL6i, respectively) (Supp. Figure 1). Two silencing sequences were evaluated against IL-6 to account for off-target effects. As shown in Fig. 2A,B, the tested sequences significantly reduced the quantity of *IL-6* mRNA and secretion, respectively. Both sequences were tested in the majority of the experiments, although for brevity only one sequence is shown in the figure panels (IL-6ia). We next analyzed whether inhibition of IL-6 expression impacted the capacity of hMSCs to regulate T-cell proliferation. As expected, hMSC-emp significantly impaired T-cell proliferation in a dose-dependent manner (Fig. 2C). By contrast, activated T-cells cultured with hMSC-IL-6i proliferated significantly greater than those cultured with hMSC-emp (Fig. 2C).

We next determined whether these differences were due to changes in the MSC:PBMC ratio. Analysis of spontaneous cell death measured by Annexin V revealed no significant differences between hMSC-IL-6i and control hMSC-emp, not even in the presence of exogenous IL-6 (20 ng/ml) (Supp. Figure 2).

Loss of IL-6 expression did not modify the adherence capacity of hMSCs, as the number of adhered cells did not change 24 h after plating (data not shown). Thus, we excluded differences in basal apoptosis or adherence capacity as a cause for the evident differences in the immunoregulatory capacity of hMSC-IL6i.

We next reasoned that the addition of exogenous IL-6 to cell cultures should be able to recover the loss of immunoregulatory capacity observed in hMSC-IL6i. Surprisingly, however, the addition of rhIL-6 to the cultures failed to reverse the immunosuppressive phenotype of hMSC-IL-6i (Fig. 2D). Moreover, inactivating extracellular IL-6 using a specific neutralizing anti-IL6 antibody^26,27^ in non-transduced hMSC cultures had no effect on the immunosuppressive process, as lymphocyte proliferation was unchanged as compared with cultures in the absence of the anti-IL6 antibody (Fig. 2E). Overall, these experiments suggest that IL-6 does not play a canonical extracellular autocrine signaling role in this cell model.

### Basal prostaglandin E2 secretion is enhanced in hMSC-IL-6i

**Figure 3:**
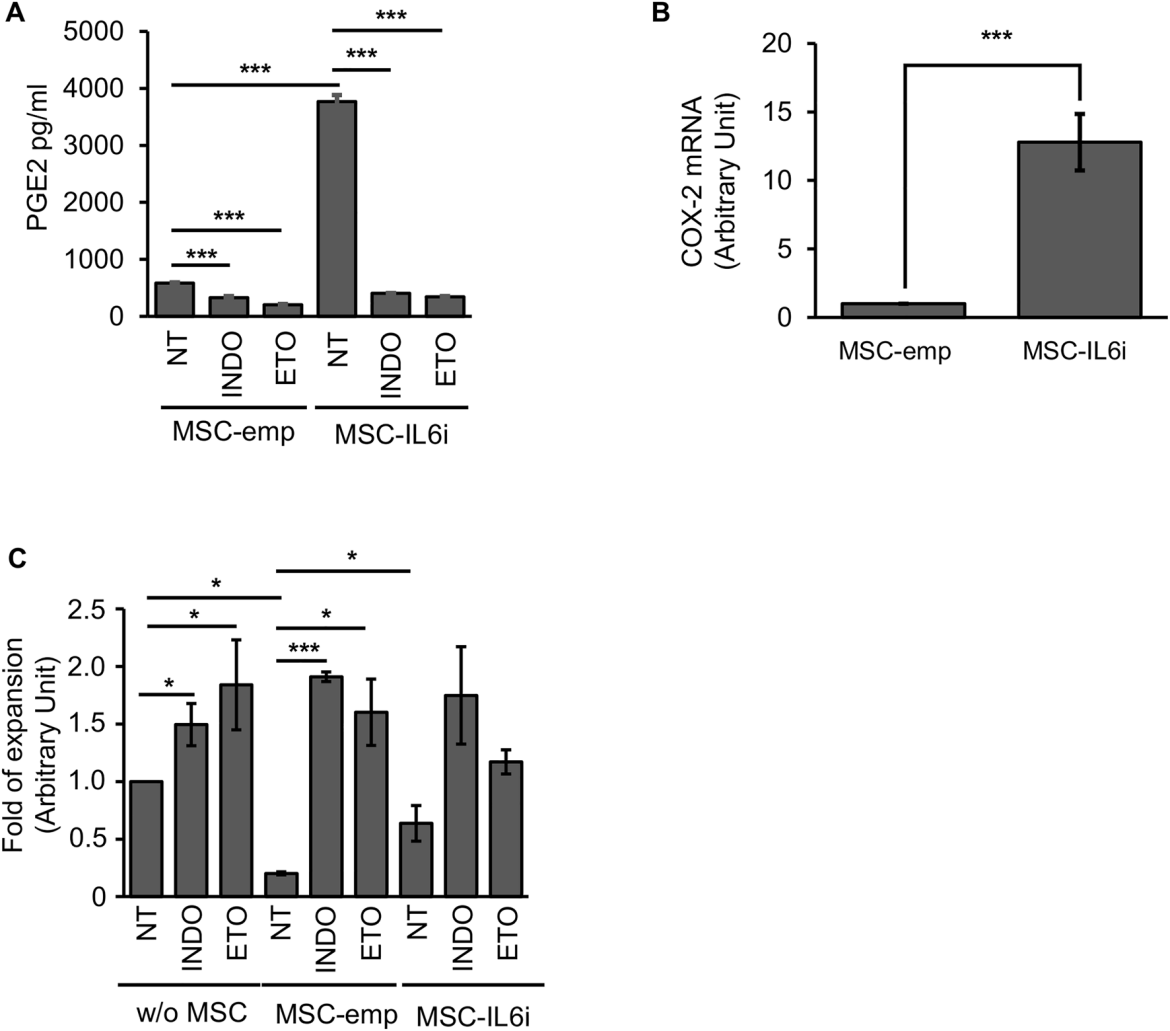
PGE2 secretion is elevated in hMSC-IL6i cells. (**A**) Secretion of PGE2 into cell culture supernatants of hMSC-emp or hMSC-IL6i, treated or not (NT) with indomethacin (INDO) or etoricoxib (ETO) (5 μM), measured by ELISA. (**B**) Quantification of *COX2* mRNA by real time qPCR in hMSC-emp or hMSC-IL6i cells. Data are shown as mean ± SD from three independent experiments. (**C**) Proliferation of T-cells cultured in the absence of hMSCs (w/o) or co-cultured with hMSC-emp or hMSC-IL6i cells, in presence or absence (NT) of indomethacin (INDO) and etoricoxib (ETO). Data are shown as mean ± SD from three independent experiments.

Among the mechanisms proposed to mediate the immunosuppressive function of MSCs, cyclooxygenase-2 (COX-2) activity, through PGE2 production, is consistently reported as one of the most important mediators^6,28,29^. As the production of IL-6 is known to be differentially regulated by PGE2 in various cell types^30,31^, we next investigated PGE2 synthesis in hMSC-IL6i cells. As shown in Fig. 3A, basal levels of PGE2 were significantly higher in hMSC-IL6i than in control cells, correlating with a significant up-regulation of *COX2* expression (Fig. 3B), and suggesting that hMSCs have a mechanism to control constitutive IL-6 expression by PGE2. The specificity of the PGE2-secretion was confirmed by blocking its production with indomethacin (a non-selective COX inhibitor)^32^ or etoricoxib (a specific COX-2 inhibitor), which induced a decrease of basal PGE2 in hMSC-IL6i, reaching the level of control cells (Fig. 3A). These results suggest an increase of basal COX-2 activity in hMSC mediated by the reduction of IL-6 levels (hMSC-IL6i). However, when we examined the immunoregulatory capacity of hMSCs after treatment with COX-2 inhibitors, we observed that in both control and hMSC-IL6i cells, treatment with COX-2 inhibitors induced a similar decrease in immunoregulatory capacity (Fig. 3C). Overall, these data indicate that PGE2 is not related to the impairment of the immunosuppressive capacity of hMSC-IL6i.

### Importance of IL-6 in the cell cycle progression

**Figure 4:**
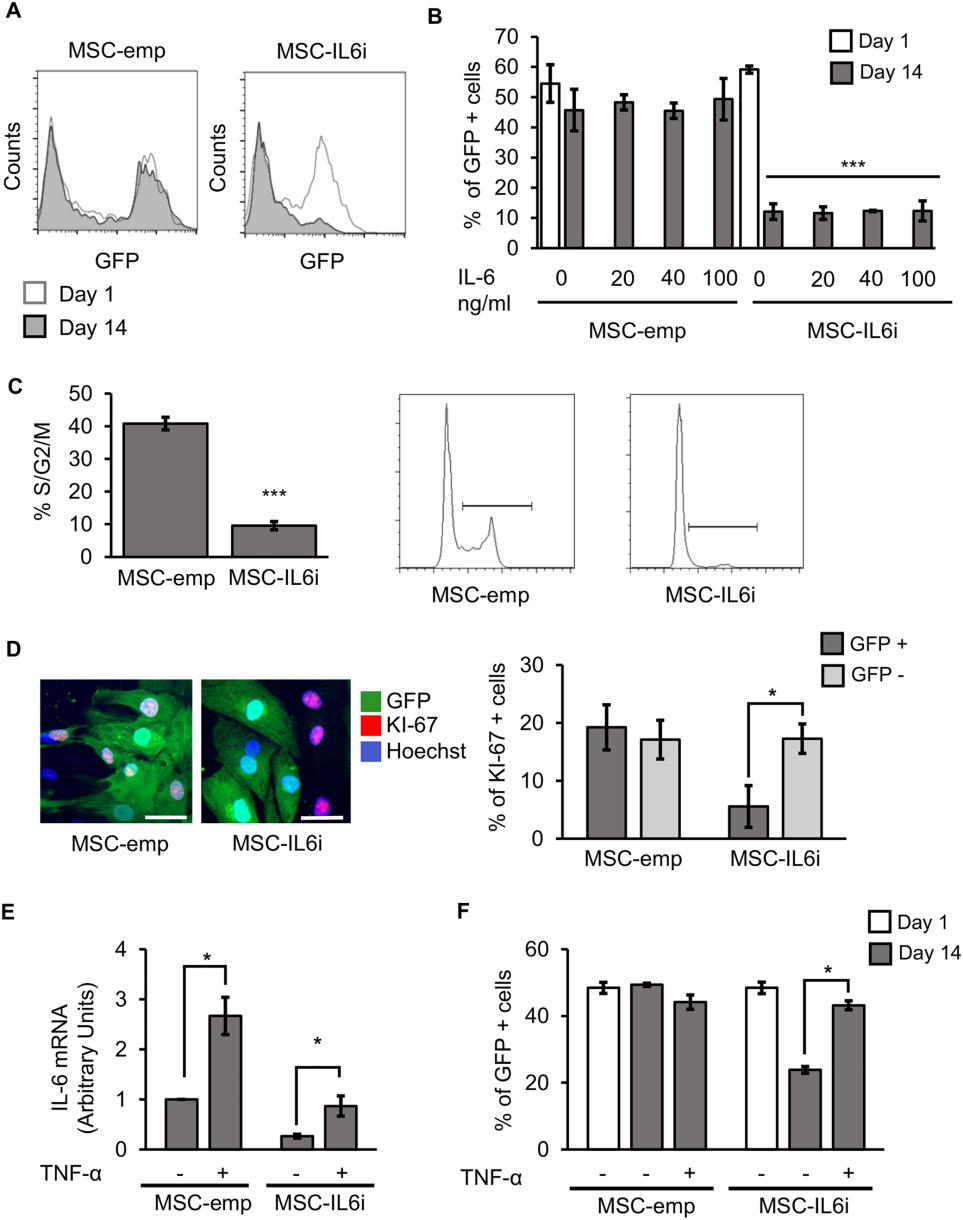
IL-6 silencing blocks cell division. (**A**) Representative flow cytometry analysis of hMSC-emp or hMSC-IL6i cells. (**B**) Percentage of GFP+ cells transduced in hMSC-emp or hMSC-IL6i cells, maintained in culture for 1 or 14 days in presence of different concentrations of recombinant IL-6. The histogram shows the mean and SD of 3 independent experiments. White bars % of GFP+ cells at day 1; dark grey bars % of GFP+ cells at day 14. ***p < 0.001. (**C**). Representative flow cytometry analysis of DNA quantification and quantification of DNA content of hMSC-emp or hMSC-IL6i cells, stained with propidium iodide and analyzed by flow cytometry. ***p < 0.001. (**D**) Representative images of the immunodetection of KI-67 in hMSC-emp or hMSC-IL6i cells. Green: GFP; Red: KI-67; Blue: Hoechst 33,258. The scale represents 50 μm (left panel). Quantification of % of hMSC KI-67+ in different cell subpopulations of hMSC-emp or hMSC-IL6i cultures. Dark grey: hMSC GFP+ . Light Grey: hMSC GFP− . *p < 0.05. (**E**) Increasing the intracellular levels of IL-6 by TNF-α stimulation recovers hMSC-IL6i proliferation. Quantification of *IL-6* mRNA expression in different hMSC populations treated ( +) or not ( −) with TNF-α (15 ng/ml). The expression levels of the target gene were normalized against *GAPDH* expression. (**F**) Percentage of GFP+ hMSC-emp or hMSC-IL6i cells treated with standard medium ( −) or standard medium supplemented with TNF-α ( +) (15 ng/ml) for 2 weeks. White bars: % of GFP+ cells at day 1. Dark grey bars: % of GFP+ cells at day 14. *p < 0.05.

We next evaluated the effect of IL-6 on hMSC proliferation. To do this, 50% of hMSCs were transduced with the empty or IL6i viral vector and then re-plated at low density (500 cells/cm^2)^, and cell growth rate was determined by analysis of the ratio of GFP+ to GFP- cells in the culture (Fig. 4A). As expected, hMSC-emp maintained the same ratio of GFP expression along the culture period of 14 days, indicating that viral integration had no effect on cell proliferation. By contrast, hMSC-IL6i failed to increase in number, which resulted in an overgrowth of non-transduced cells at confluence (Fig. 4A). This phenotype was unaffected by the presence of different concentrations of rhIL-6 (Fig. 4B). The finding that the unmodified hMSCs are able to overgrow hMSC-IL-6i even in the presence of high concentrations of rhIL-6 suggests that the proliferation of hMSCs is dependent of intracellular rather than extracellular IL-6. To confirm this, we analyzed the effect of IL-6 silencing on the cell cycle. Flow cytometry analysis showed that the percentage of cells in the active phases of the cell cycle (S/G2/M) was significantly lower for hMSC-IL-6i than for control cells, and the cell cycle was mainly blocked in G0/G1 in the former (Fig. 4C). These results suggest that the loss of IL-6 expression in hMSCs not only affects immunosuppression, but also impairs their normal proliferation. To explore this further, we analyzed KI-67 expression in hMSC-emp and hMSC-IL6i. KI-67 protein is present almost exclusively in the G1/S/G2/M phases, and is a very useful marker for recognizing dividing cells. As before, 50% of the hMSCs were transduced with the silencing/control vectors and the expression of KI-67 was analyzed by immunofluorescence in GFP+ and GFP- cells (Fig. 4D). We failed to observe differences in KI-67 expression between GFP- cells of hMSC-emp and hMSC-IL6i. However, we observed a significant decrease in KI-67 expression in GFP+ hMSC-IL6i cells, confirming the defect in proliferation. These results highlight the inability of hMSC-IL6i to progress through the cell cycle and this phenomenon does not involve extracellular IL-6, as all cells (both GFP+ and GFP-) were in the presence of the same amount of soluble IL-6 and only hMSC-IL6i cells showed impaired proliferation. Finally, we stimulated hMSC-IL6i with TNF-α (15 ng/mL) aiming to drive IL-6 expression (Fig. 4E). We found that this treatment allowed a significant recovery of hMSC-IL6i proliferation (Fig. 4F).

### IL-6 silencing in hMSC-IL6i cells impedes ERK1/2/cyclin D1 pathway signaling

**Figure 5:**
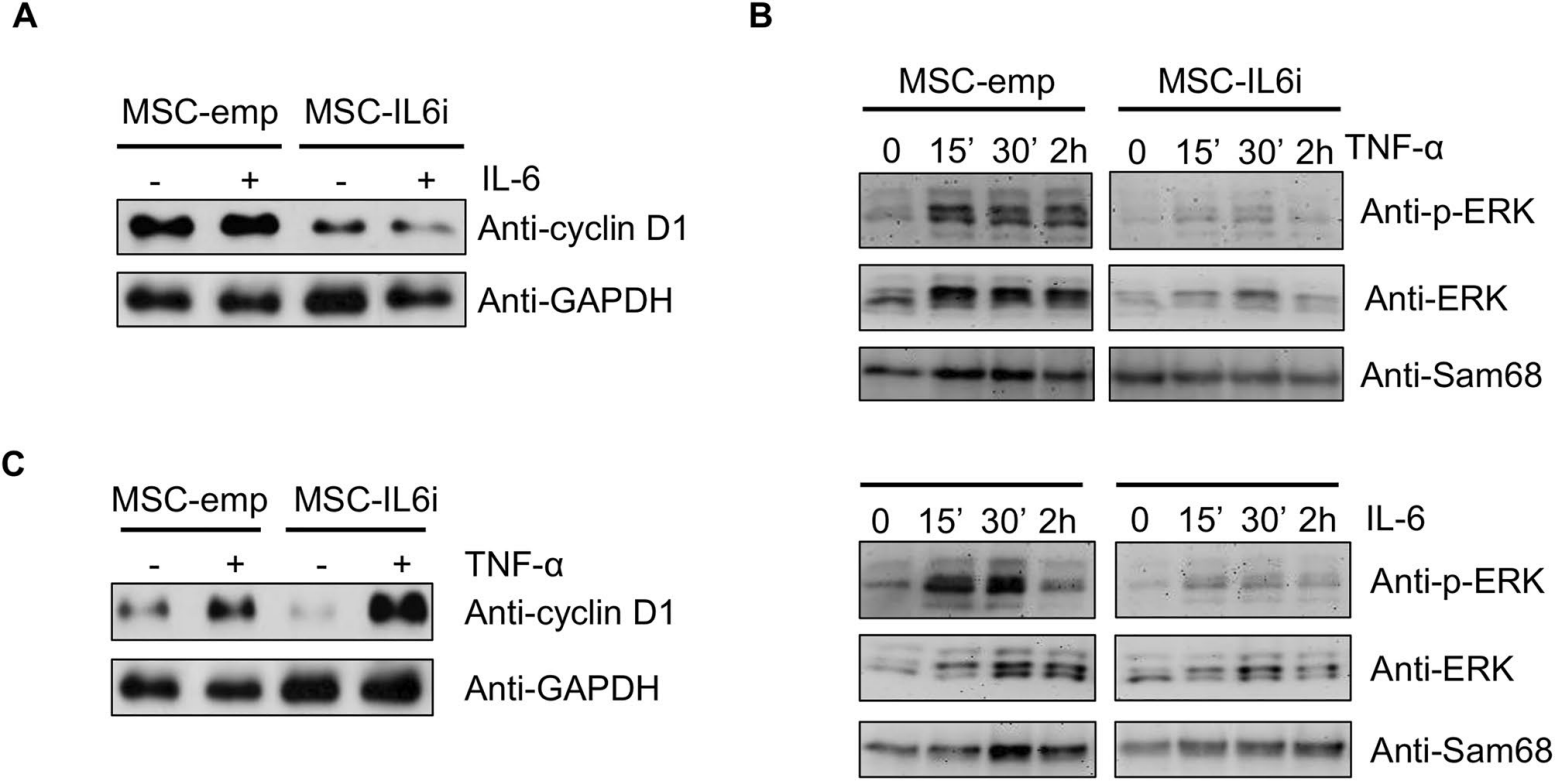
IL-6 silencing blocks cell division through a decrease of cyclin D1 and ERK1/2phosphorylation. (**A**) Cyclin D1 expression was analyzed in hMSC-emp or hMSC-IL6i cells treated ( +) or not ( −) with IL-6 (20 ng/ml). GAPDH was used as a loading control (**B**) hMSC-emp or hMSC-IL6i cells were treated for the indicated times with TNF-α (15 ng/ml) (upper panel) or IL-6 (20 ng/ml) (lower panel) to analyze the level of phospho-ERK1/2. Sam68 was used as a loading control. (**C**) Cyclin D1 expression in hMSC-emp or hMSC-IL-6i cells treated ( +) or non-treated ( −) with TNF-α (15 ng/ml) for 48 h. GAPDH was used as a loading control.

Cyclin D1 is involved in the control of the cell cycle G0/G1/S transition and a defect in its expression in hMSCs alters the normal process of cell division^33^. Given our results, we next analyzed cyclin D1 levels in hMSC-IL6i cells by western blotting. As shown in Fig. 5A, the level of cyclin D1 was significantly lower in hMSC-IL6i than in hMSC-emp. Remarkably, the addition of rhIL-6 (20 ng/ml) to the culture medium failed to recover cyclin D1 expression (Fig. 5A). We previously demonstrated that ERK2 (extracellular signal-regulated kinase 2) was a key transcription factor in the proliferation of hMSC, partly through its control of cyclin D1 expression^33^. To analyze the activation state of ERK2, we monitored the levels of ERK1/2 phosphorylation in hMSC-emp and hMSC-IL6i in the presence or not of rhIL-6 (20 ng/ml) or TNF-α (15 ng/ml). Results shown in Fig. 5B demonstrate a decrease of ERK2 phosphorylation in hMSC-IL6i, in both conditions. To confirm that intracellular IL-6 plays a critical role in hMSC proliferation through cyclin D1, we investigated whether the increase in IL-6 stimulated by TNFα in hMSC-IL6i (see Fig. 1) was capable of recovering cyclin D1 levels. We observed a very significant increase in cyclin D1 expression in cells stimulated for 48 h with TNF-α (Fig. 5C), which was in sharp contrast to the failure of exogenous rhIL-6 to modify cyclin D1 level (Fig. 5A). These data allow us to conclude that IL-6 is related to the control of cell cycle progression through ERK1/2 and by an exclusively intracellular signaling pathway.

## Discussion

hMSCs possess immunosuppressive capabilities, which endow them potential to treat inflammatory diseases^1^. hMSCs have capacity to regulate many aspects of T-cell response, such as proliferation, survival and differentiation. Numerous molecules secreted by hMSC and/or T-cells are known to be been involved in the regulation of hMSC immunoregulation^1^; however, the controlling mechanisms are not fully understood. We recently described the importance of the NF-κB pathway in hMSC physiology. Here, we investigated the possible involvement of IL-6, a pleiotropic cytokine whose expression is under control of NF-κB transcription factor, in hMSC immunoregulation. Using a gene silencing approach, we show that the inhibition of IL-6 expression significantly impairs hMSC immunoregulatory functions. Interestingly, the effect of IL-6 is not due to its secretion into the extracellular milieu, since the addition of rhIL-6 (even concentrations up to 100 ng/ml) to the cultures could not reverse the observed phenotype.

Numerous studies have investigated the involvement of soluble factors in the regulation of hMSC function, of which PGE2 is consistently described as one of the most important^34^. Accordingly, we were intrigued by the unexpected results showing that PGE2 secretion was consistently elevated in hMSC-IL6i cells, particularly since it has been described that the immunomodulatory function of hMSCs is partially attributed to IL-6-dependent secretion of PGE2, most likely through the positive regulation of COX-2 activity by IL-6^35^. Indeed, PGE2 secretion was significantly reduced in IL-6-deficient MSCs, which translated into a poor immunosuppressive ability in a collagen-induced experimental arthritis mouse model^35^. The discrepancy between these observations and ours is most likely due to the differences in the MSCs used. Moreover, in the aforementioned study by Bouffi et al^35^, C57BL/6 mice deficient for IL-6 were used, whereas in our study the expression of IL-6 was considerably reduced but not abolished. Several works have also implicated PGE2 in IL-6 production^36,37^. Therefore, we can hypothesize that the hMSC-IL6i cells, through a mechanism that remains to be determined, attempt to compensate for the loss or reduction in IL-6 expression by increasing COX2 expression and PGE2 secretion. In this line, we consistently observed a slight increase in NF-κB activity in hMSC-IL6i cells (data not shown). Nevertheless, these compensatory mechanisms do not seem to be sufficient to restore IL-6 expression and recover the immunoregulatory function of hMSCs. Further work will be necessary to determine the controlling mechanisms in the increase of PGE2 secretion.

Our experiments demonstrate the involvement of IL-6 in the control of hMSC proliferation through an intracellular mechanism. As far as we know, this phenomenon has never been described in primary cell lines although other groups have shown similar phenotypes in tumor-derived cell lines including renal carcinoma^38^, choriocarcinoma^20^ and melanoma^39^, in which reduced IL-6 expression slows their proliferation, whereas blocking IL-6 or gp-130 using specific antibodies do not affect cellular growth. We found that in hMSCs this proliferative effect is due to a defect of cell cycle progression that is rescued by partially recovering the levels of intracellular IL-6 expression through stimulation with TNF-α. In a previous study, we demonstrated that ERK2 is a key transcription factor in the proliferation of hMSCs, in part through its control of cyclin D1 transcription and, therefore, expression^33^. Cyclin D1 is a positive regulator of the cell cycle and promotes G1 to S phase transition in cooperation with CDK4 or 6. Since the protein level of cyclin D1 reflects cell cycle progression, the rates of protein production and degradation are strictly regulated at the level of the transcription and protein degradation^40^. Indeed, cyclin D1 is highly labile, with a half-life of 10–30 min, and undergoes polyubiquitination and proteasomal degradation^40^. Here, we demonstrate that in the absence of IL-6, the level of cyclin D1 is significantly decreased. This might also be due, at least in part, to inhibition of the MAPK and/or AKT pathways. Further work will be necessary to understand how the loss of IL-6 induces a decrease of ERK1/2 phosphorylation in hMSCs.

Overall, our findings reveal that IL-6 is a pivotal factor in the proliferation of hMSCs, but highlight that its effects occur at the intracellular and not at the extracellular level. Other studies have suggested that IL-6 could act as an autocrine/intracrine growth factor interacting with its specific receptors within the cell and not at the cell surface^20,38,41^. More work is needed to identify the intracellular receptors for IL-6. In this regard, our preliminary experiments suggest that small quantities of IL-6 receptor are present in the cytoplasm of some cells (data not shown). If confirmed, IL-6 may activate different transduction pathways through an interaction with its receptor in intracellular compartments. Our results open the door for further research into whether other interleukins can signal without the need for secretion, which would be a considerable advance in understanding the autocrine mechanisms of cellular regulation.

## Conclusions

IL-6 is synthesized and secreted by many cell types including human mesenchymal stromal cells. It is often referred to as a pleiotropic cytokine that influences numerous cell types, with multiple biological activities. In this study, we have examined the role of this interleukin in the homeostasis of hMSC. Our results show that IL-6 is essential for the proliferation of stromal cells and their immunosuppression capacity. Nevertheless, our most relevant finding relates to a previously undescribed signaling mechanism of IL-6-driven MSC homeostasis. Our data show that changes to the extracellular levels of IL-6 do not resemble the phenotype observed when modifying intracellular expression, indicating that IL-6 signals intracellularly in hMSC. Further research is necessary to integrate the intracellular signaling described in this study with current knowledge on the role of this interleukin in vivo. Incorporating a new signaling mechanism into the understanding of IL-6 (or other interleukins) biology will be a huge step forward in unravelling cytokine-mediated intercommunication in human biology.

## Supplementary Information

Supplementary Information

## Data Availability

All data generated and/or analyzed during this study are included in this published article and its Additional files.

## Abbreviations

CBA: Cytometric bead array
CDK: Cyclin dependent kinases
COX-2: Cyclooxygenase 2
ERK: Extracellular signal-regulated kinases
ETO: Etoricoxib
GAPDH: Glyceraldehyde-3-phophate dehydrogenase
GFP: Green fluorescent protein
gp-130: Glycoprotein 130
hMSC: Human mesenchymal stromal cells
IDO: Indoleamine 2,3-dioxygenase
IL-1 RA: Interleukin-1 recepetor antagonist
IL-10: Interleukin-10
IL-6: Interleukin-6
IL-6R: Interleukin-6 receptor
INDO: Indomethacin
mRNA: Messenger RNA
NF-κB: Nuclear factor kappa-light-chain enhancer of activated B cells
PBMC: Peripheral blood mononuclear cells
PGE2: Prostaglandin E2
PI: Propidium iodide
rhIL-6: Recombinant human interleukin-6
SDS–PAGE: SDS–polyacrylamide gel
shRNA: Short hairpin RNA
TNF-α: Tumour necrosis factor alpha
