# Supplementary material for: Intracellular role of IL-6 in mesenchymal stromal cell immunosuppression and proliferation
Source: Sci Rep. 2020 Dec 14;10:21853. doi: 10.1038/s41598-020-78864-4 (PMC7736882; doi:10.1038/s41598-020-78864-4)
Supplement: Supplementary file 1 — Supplementary Information [file 41598_2020_78864_MOESM1_ESM.pdf]

Title: Intracellular role of IL-6 in mesenchymal stromal cell immunosuppression and proliferation.

Authors: Akaitz Dorronsoro[1,2*†], Valérie Lang[1*], Izaskun Ferrin[1], Jon Fernández-Rueda[1], Lorea Zabaleta[1], Estibaliz Pérez-Ruiz[1], Pilar Sepulveda[2], César Trigueros[1]

[1]Fundación Inbiomed, Foundation for Stem Cell Research, Mesenchymal Stem Cell Laboratory, Paseo Mikeletegi, 81. 20009. San Sebastián, Spain.

[2]Instituto de Investigación Sanitaria La Fe, Regenerative Medicine and Heart Transplantation Unit, Valencia, Spain., Avenida Fernando Abril Martorell, 106. Torre A, Laboratorio 5.04, 46026 València, Spain

Running Title: IL-6 signals intracellularly in hMSCs.

Key words: IL-6, hMSCs, T cells, immunosuppression, proliferation, intracellular.

***: Equal participation**

†: To whom correspondence should be addressed:

E-mail: akaitz82@gmail.com
Akaitz Dorronsoro, Ph.D.
Avenida. Fernando Abril Martorell, 106.
Torre A, Laboratorio 5.04. 46026
Valencia, Spain.
Tlf: +34 961246632

Supplementary Figure 1

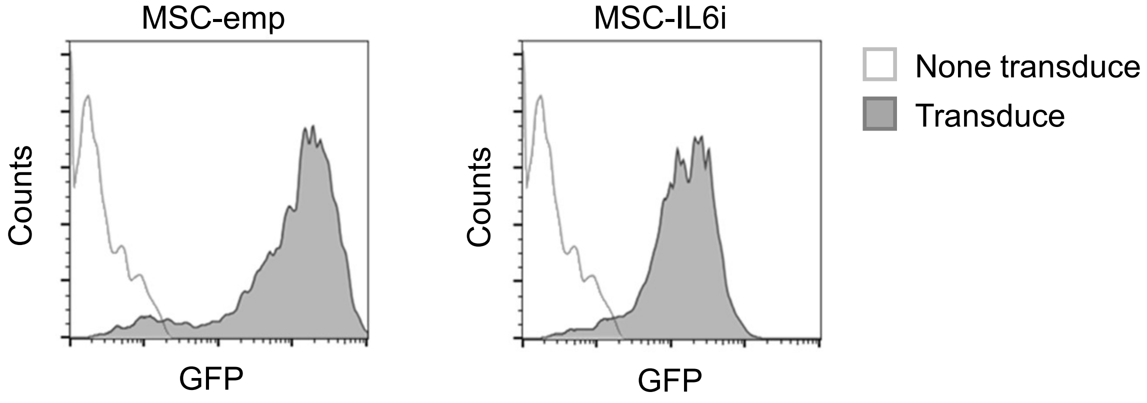

Supplementary Figure 2

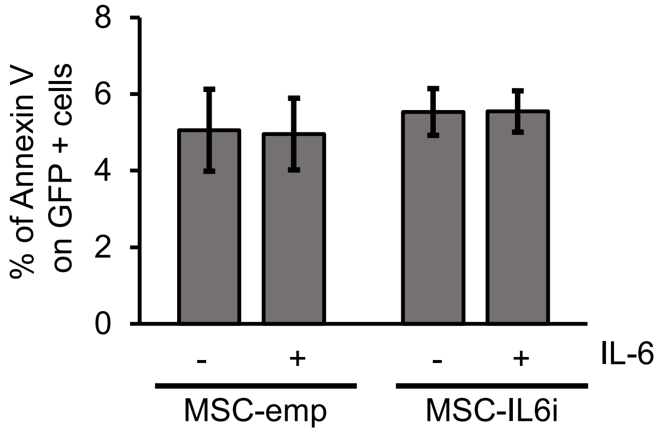

Supplementary Figure 3

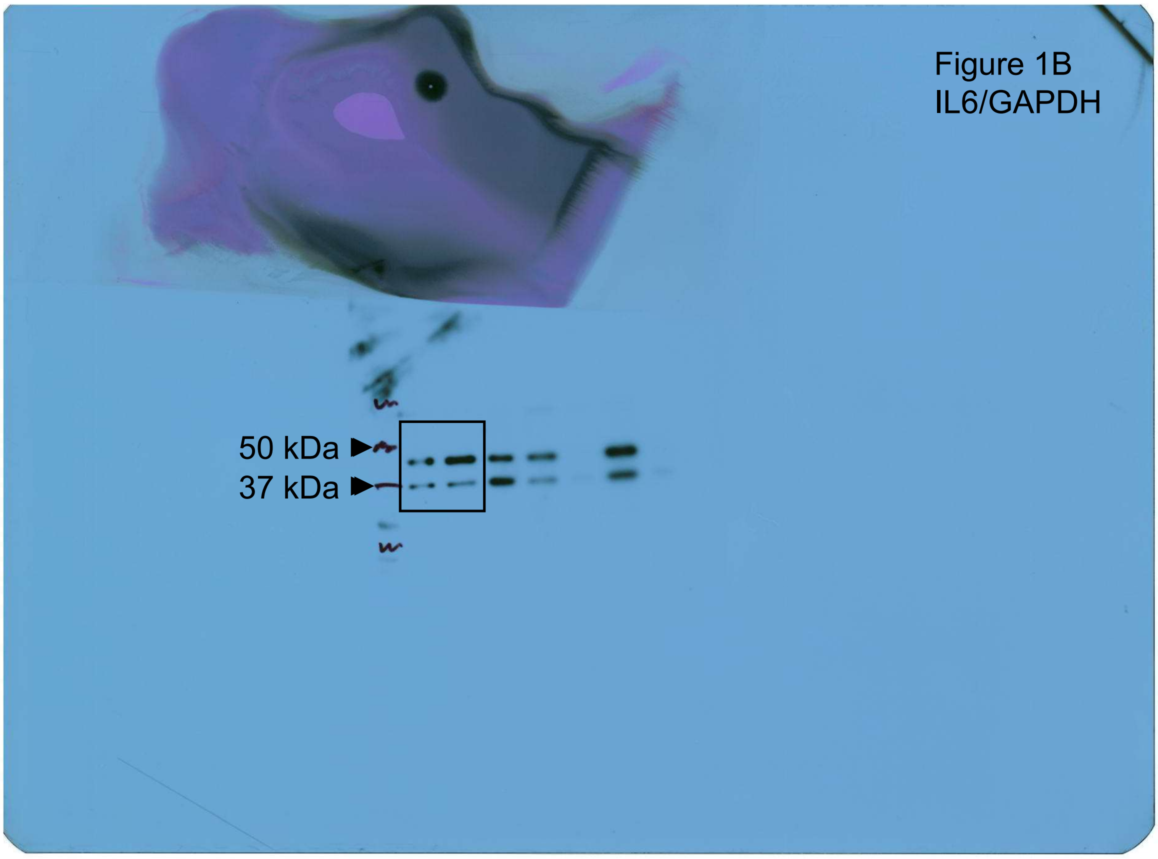

Supplementary Figure 4

A

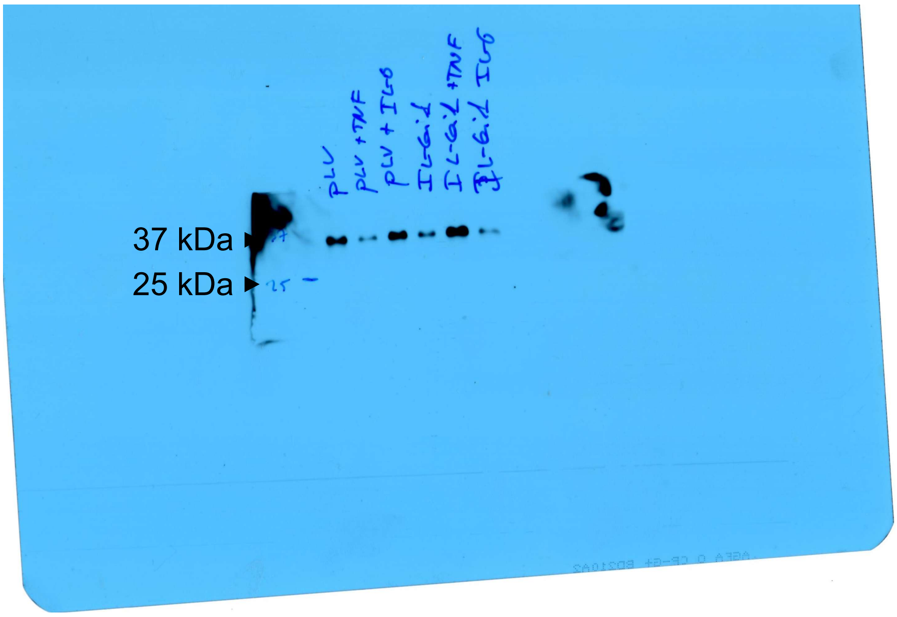

Figure 5A
Cyclin D1

B

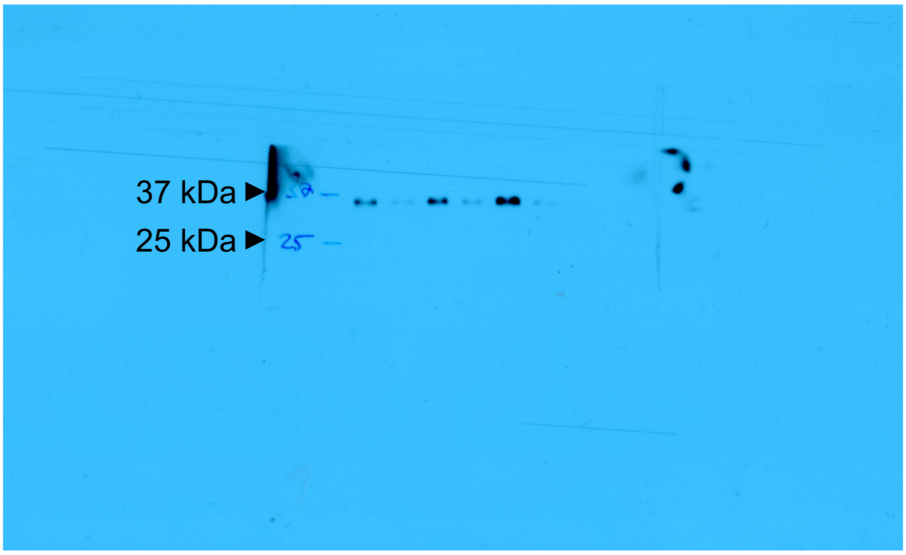

Figure 5C
Cyclin D1

C

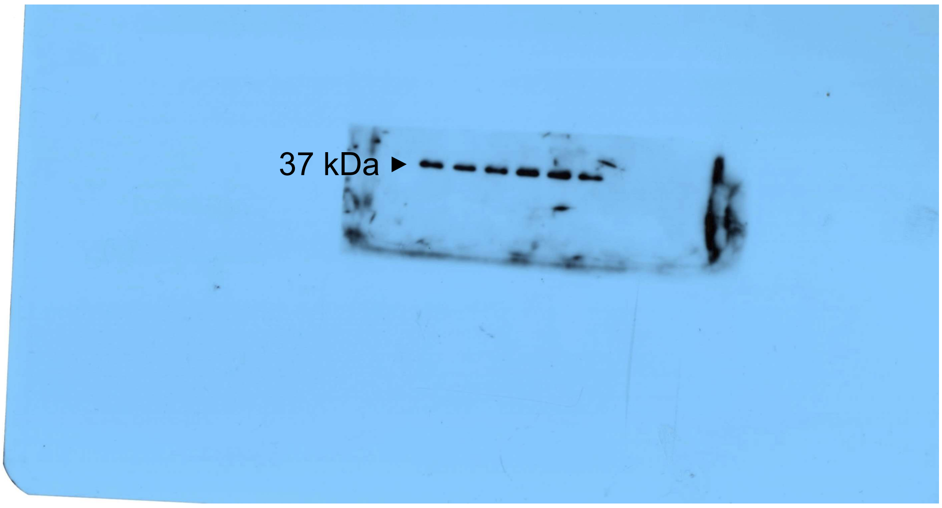

Figure 5 A/C
GAPDH

Supplementary Figure 5

A

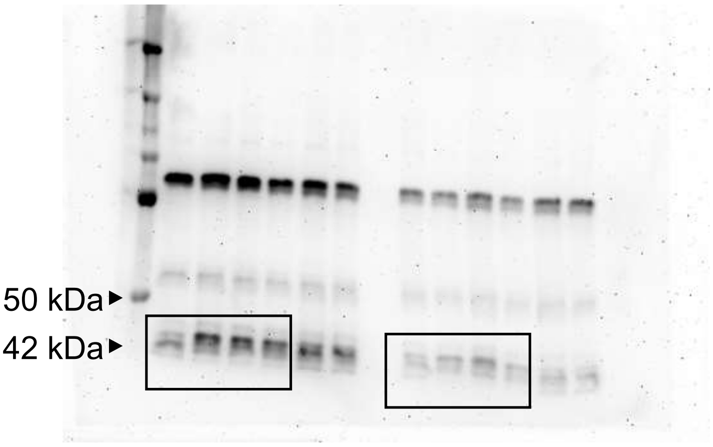

Anti ERK

B

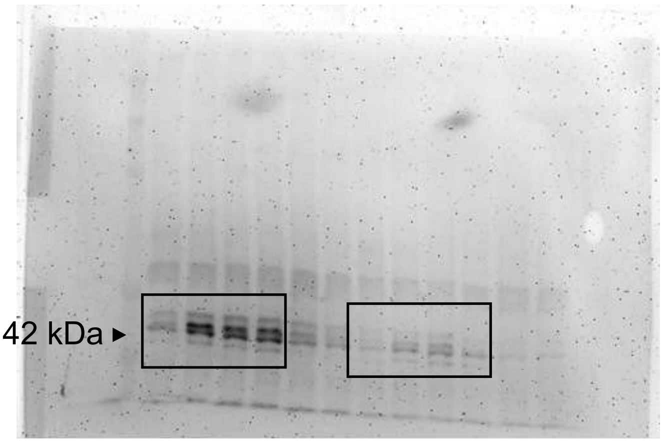

Anti p-ERK

C

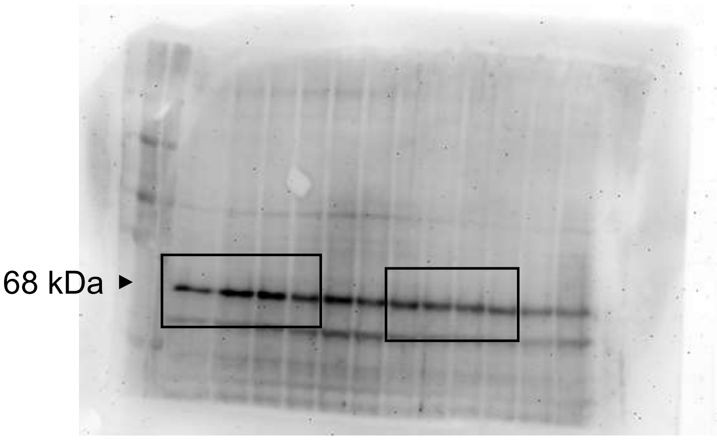

Anti Sam68

Figure 5B, upper panel

Supplementary Figure 6

A

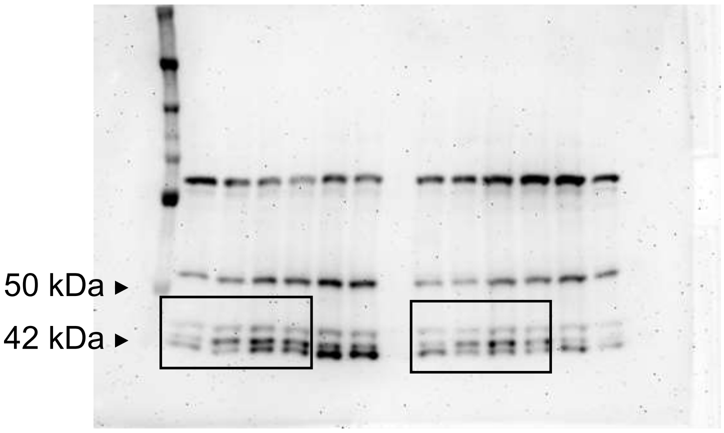

Anti ERK

B

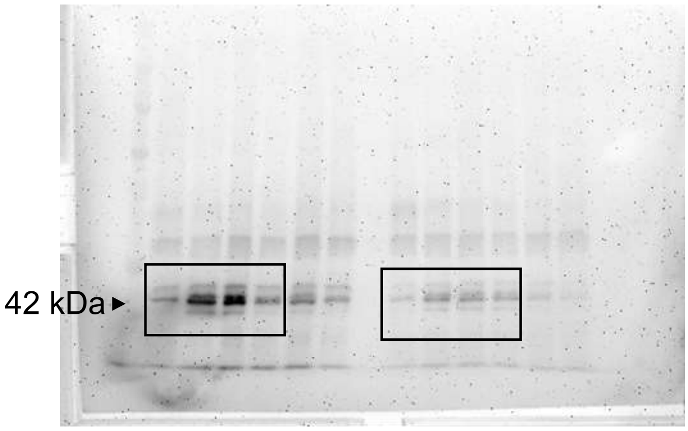

Anti p-ERK

C

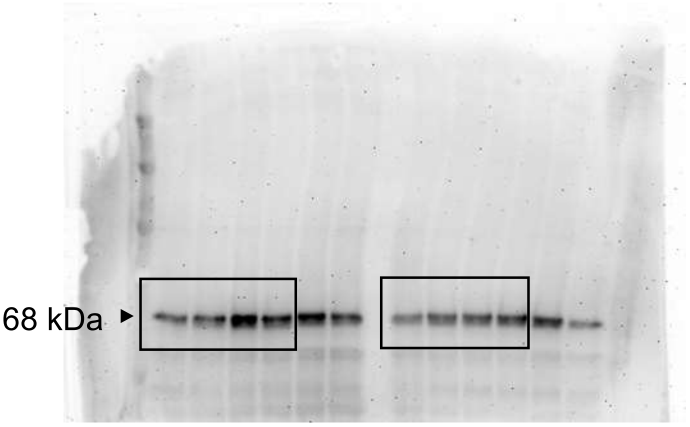

Anti Sam68

Figure 5B,
lower panel

SUPPLEMENTARY FIGURE LEGENDS

Supplementary figure 1. **Representative flow cytometry analysis of hMSC-emp or hMSC-IL6i cells transfected at <95%.** Empty histograms are untransduced MSCs. Filled grey histograms are transduced MSCs.

Supplementary figure 2. **IL-6 silencing does not trigger cell death.** hMSC-emp or hMSC-IL-6i cells were cultured overnight in standard medium (-) or supplemented with IL-6 (20 ng/ml). Apoptosis was analyzed by FACS by Annexin-V DY634 and propidium iodide (PI) staining (the percentage of Annexin-V positive/PI negative cells is shown) on GFP + cells. Graph represents the mean of two independent experiments.

Supplementary figure 3. **Uncropped blot image of bands shown in Figure 1 B.** The cropped bands are framed within the black square.

Supplementary figure 4. **Uncropped blot image of bands shown in Figure 5A and 5C.** Handwritten tags indicate the sample loaded in each lane. MSC-emp (plv) & IL6i1 (MSC-IL6i)

Supplementary figure 5. **Uncropped blot image of bands showen in figure 5B, upper panel.** The cropped bands are framed within the black square.

Supplementary figure 6. **Uncropped blot image of bands shown in Figure 5B, lower panel.** The cropped bands are framed within the black square.
